# The Effect of Emotional Reactivity on Marital Quality in Chinese Couples: The Mediating Role of Perceived Partner Responsiveness

**DOI:** 10.3389/fpsyg.2021.787899

**Published:** 2022-01-18

**Authors:** Qunming Yuan, Zhiguang Fan, Jiaqi Leng

**Affiliations:** School of Education, Jilin International Studies University, Changchun, China

**Keywords:** emotional reactivity, marital quality, perceived partner responsiveness, actor-partner interdependence model (APIM), Chinese couples

## Abstract

This study investigated the mediating role of perceived partner responsiveness in the relationship between emotional reactivity and marital quality among Chinese couples. The survey participants included 550 couples from 28 provinces in the Eastern, Central and Western China. The ages of the husbands range from 39 to 64 years old whose average age is 46.45 years old, while the ages of the wives vary between 32 and 62 years old whose average age is 45.08 years old. The Emotion Reactivity Scale, Perceived Partner Responsiveness Scale, and Quality of Marriage Index were selected for measurement. The results showed that the scores of husbands perceived partner responsiveness and marital quality were significantly higher than those of wives, and there was no significant difference in emotional reactivity between husbands and wives. Correlation analysis showed that emotional reactivity of couples was negatively correlated with perceived partner responsiveness and marital quality, while perceived partner responsiveness was positively correlated with marital quality. Based on the Actor-Partner Interdependence Model (APIM), it was found that the emotional reactivity of both spouses was a significant negative predictor of their marital quality (actor effect). It also significantly negatively predicted the marital quality of the spouse (partner effect). The mediating effect analysis results showed that the husbands’ perceived partner responsiveness played a mediating role in the emotional reactivity of the couples on marital quality of the husbands, and the wives’ perceived partner responsiveness played a mediating role in the emotional reactivity of the couples on marital quality of the husbands. The wives’ perceived partner responsiveness played a mediating role in the effect of the couples’ emotional reactivity on the wives’ marital quality. The results of this study contribute to a better understanding of the mechanism of emotional reactivity of couples affecting marital quality and have a guiding significance for improving marital quality.

## Introduction

For most people, marriage is one of the most important issues that need to be faced and resolved in a person’s life. Family originated from marriage. Parent-child relationship and sibling relationship are all developed on the basis of marriage relationship. Marital quality can affect the harmony of family members, which is a prerequisite for family functions to be exerted (Yoo, 2020).

The research on marital quality has always been a hot topic in the field of marriage and family. In Confucianism’s opinion, marriage is the beginning of human relations as well as the basis of policy and kingdomlization. In addition, in the traditional Chinese concept of marriage, the children’s marriage is entirely decided by their parents, and the couple have not met each other even once before getting married. In the past 20 years, with the rapid development of China’s economy, the continuous improvement of people’s educational level, the continuous development of urbanization, and the impact of multiculturalism, the traditional view of marriage and family has changed (Mo, 2020). People’s expectations for marriage are higher and higher, and they also pay more attention to marital quality (Su et al., 2018).

Marital quality is the subjective evaluation of the marital relationship by both spouses, and it is the key factor that affects marital happiness and life quality (Fowers and Owenz, 2010). Previous studies have shown that the marital quality is closely related to the loneliness and infidelity of married adults (Isanejad and Bagheri, 2018). Low-quality marriages tend to have strong instability which has a good predictive effect on divorce tendency (James, 2015). In addition, the marital quality of parents can also affect the psychological and behavioral development of their children. On the one hand, cross-sectional studies have found that the parental marital quality is related to the occurrence of children’s anxiety, fear, depression, aggression (Gamliel et al., 2018), social withdrawal, and eating disorders and other behavioral problems and emotional distress (Salafia et al., 2016). On the other hand, follow-up studies have also confirmed that the marital quality of parents can also affect their children’s future development (Rhoades et al., 2011) and mental health (Ding et al., 2019). Therefore, it is of great significance to explore the influencing factors of marital quality and further reveal its influencing mechanism to maintain the harmonious coexistence of families.

### The Direct Effect of Emotional Reactivity on Marital Quality

The establishment of marriage means that the couple needs to face and solve more negative life events, such as conflicts and differences between the two parties (Ali et al., 2020), work-family conflicts, economic pressure, children’s education, etc. (Ying et al., 2020). The negative emotions caused by negative events are not only not conducive to problem solving, but also have a negative impact on the development of the marital relationship and the marital quality (Frye et al., 2020). It is worth noting that individuals with different emotional response characteristics may produce different results when facing the same pressure (O’Connor et al., 2018). Emotional reactivity is the emotional experience generated by an individual under the action of the evoked stimulus, the intensity of the perceived emotion, and the time required for the emotion to return to the baseline level (Nock et al., 2008). Emotional reactivity includes three aspects: emotional sensitivity, emotional intensity and emotional persistence. Individuals with high emotional reactivity have difficulty in emotional coping and emotion regulation. When they face negative stimuli, they will show high sensitivity, high intensity and slow recovery. The higher a person’s emotional reactivity level is, the lower the threshold of emotional arousal will be. In the same stress situation, individuals with high emotional reactivity level will have stronger emotional reactivity, longer duration, and more likely to lead to mood disorders, depression disorders (Shapero et al., 2016), anxiety disorders and other mental disorders (Dell et al., 2020). In addition, the stronger the individual’s emotional reactivity and the more difficult it is to regulate emotions, the easier it is to experience anxiety, pain, fear, anger, jealousy (Chapman et al., 2015), disgust and other emotional responses (Polanco-Roman et al., 2018). Therefore, high emotional reactivity has a damaging effect on marital quality.

### The Actor-Partner Interdependence Model of Emotional Reactivity on Marital Quality

Based on family system theory, as a subsystem of the family system, couples form an emotional community, which connects with each other emotionally (Davies and Cicchetti, 2004). The emotional experience of one party can be transmitted to the other party, thus becoming the problems that both husbands and wives need to deal with together (Erdem and Safi, 2018). Emotional reactivity is considered as the core index to measure the marital quality of a couple. Couples with positive emotional reactivity often support each other when facing daily pressure (Gardner et al., 2007), which helps to create a harmonious family atmosphere and thus improves the marital quality of both parties (Amber et al., 2020). What’s more, people’s attributions of their partners’ negative emotions also influence their marital quality. If individuals perceive their spouses as less emotionally reactive, they are more inclined to attribute the cause of their emotions to the external environment when they show negative emotions. That is why those individuals are more tolerant of their spouses’ negative emotions and more willing to offer support, which helps reduce marital conflict and has a positive effect on improving the quality of marriage for both partners (Ye et al., 2018). In consideration of the interaction between emotional reactivity and marital quality of couples, this study took couples as the research objects, and used Actor-Partner Interdependence Model to estimate actor effect and partner effect. The actor effect refers to the influence of individual predictor variables on outcome variables, that is, the influence of individual emotional reactivity on their marital quality. The partner effect refers to the influence of individual predictive variables on spouse’s outcome variables, that is, the influence of individual emotional reactivity on spouse’s marital quality.

### The Mediating Role of Perceived Partner Responsiveness in the Actor Effect of Emotional Reactivity on Marital Quality

Perceived partner responsiveness may play a mediation role in the actor effect of emotional reactivity on marital quality. It refers to the degree to which a person perceives that his partner pays attention to his core characteristics and responds positively (Reis et al., 2011). The individual’s emotional reactivity may have a negative impact on perceived partner responsiveness. Social regulation cycle theory holds that individuals will consciously influence others’ emotions through interpersonal interaction. In the process of couple interaction, individuals identify the spouse’s emotions, evaluate need for regulation, and decide what regulation strategies to adopt (Reeck et al., 2016). Common adjustment strategies include remaining passive, changing or avoiding. If the emotional response of the spouse is too strong, the individual tends to adopt the strategy of remaining passive, considering that it is difficult to regulate the spouse’s emotion and may even produce negative results (Reeck et al., 2016). Individuals with high emotional reaction tendencies are easy to be regarded by their spouses as unfriendly or even hostile, which reduces the frequency of their behavior reaction (Overall et al., 2020). In addition, the degree to which individuals perceive partner response is influenced by the way they respond to their partner’s needs (Lemay et al., 2007). For example, if a wife always responds positively to her husband’s needs, the wife is more likely to experience the husband’s positive response to her. Individuals with high emotional reactivity often make negative avoidance response to their spouse, which leads to a lower degree of perceived partner responsiveness.

Perceived partner responsiveness is an important factor in improving a couple’s marital quality (Alonso-Ferres et al., 2020). When individuals perceive that their spouse actively responds to their needs, desires, hobbies, etc., they will think that they are understood, cared for, and valued (Merwin and Rosen, 2020), which is conducive to the improvement of marital quality (Reuma et al., 2016). Numerous studies have shown that perceived partner responsiveness plays a decisive role in the establishment, maintenance and development of intimate relationships (Tasfiliz et al., 2018; Alonso-Ferres et al., 2021). This can be explained by the couple interaction theory and Circumplex Model of Marital and Family Systems. According to the couple interaction theory, the marital quality depends on the quantity and quality of the interaction between a couple. Positive interaction can enhance a couple’s feelings and improve the quality of marriage (Cundiff et al., 2015). Circumplex Model of Marital and Family Systems points out that the balanced couples have more positive communication skills than unbalanced couples. Good communication can promote family cohesion and flexibility. Open and positive communication among family members is conducive to the normal play of family function, so as to improve marital satisfaction (Olson et al., 2019). Therefore, perceived partner responsiveness may have a positive predictive effect on marital quality.

### The Mediating Role of Perceived Partner Responsiveness in the Partner Effect of Emotional Reactivity on Marital Quality

Emotional reactivity not only has a negative impact on individual’s perceived partner responsiveness, but also may influence the degree of spouse’s perceived partner responsiveness. Individuals with high emotional reactivity are easy to experience emotional reactions such as anxiety, pain, jealousy and disgust. They often adopt the negative communication mode of accusation and avoidance, and less self exposure. They are less willing to respond to their spouse’s needs, desires or actions (Eman et al., 2019). In addition, individuals with high emotional reactivity are often in negative emotions. They not only lack enough patience and energy to accompany their spouse, but also frequent marital conflicts may lead to negative interaction patterns, which will have a negative impact on the spouse’s perceived partner responsiveness and marital quality (Liu and Cheung, 2015). Self exposure, physical contact, sharing and mutual appreciation in the process of couple interaction can effectively promote the development of intimate relationship (Henry et al., 2007). The withdrawal behavior between husband and wife, such as silence, avoiding physical contact, being not interested in the topics raised by the spouse, and ignoring each other’s feelings, can lead to negative communication patterns. This destroys the intimacy of the family, which in turn affects the marital quality of both spouses. Therefore, perceived partner responsiveness may play a mediating role in the partner effect of emotional reactivity on marital quality.

### Objectives and Hypothesis

Marital quality is the subjective feelings of both spouses about the marriage relationship. It is affected by many factors such as social culture, stressful life events, communication styles, and personality characteristics. Among them, emotional reactivity is considered to be a key factor affecting marital quality (Gardner et al., 2007). At present, there is no research to explore the relationship between emotional reactivity and marital quality in the context of Chinese culture. In addition, previous studies focused more on the actor effect of individual emotional reactivity on marital quality. Only few studies focused on the partner effect of emotional reactivity on marital quality, especially the mediating role of perceived partner responsiveness between the two. Based on this, this study uses Chinese couples as the research object and uses the APIM model to explore the relationship between emotional reactivity, perceived partner responsiveness and marital quality, and further explore its internal mechanism.

**FIGURE 1:**
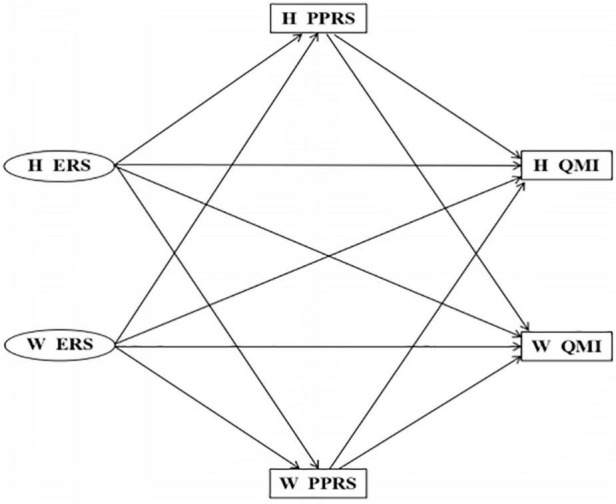
Theoretical hypothesis model. H, husbands; W, wives; ERS, emotion reactivity scale; PPRS, perceived partner responsiveness scale; QMI, quality of marriage index.

We propose the following Hypothesis (as shown in Figure 1):

H1: Emotional reactivity has a negative impact on their own marital quality (actor effect).

H2: Emotional reactivity has a negative impact on marital quality (partner effect).

H3: Perceived partner responsiveness mediates the relationship between emotional reactivity and marital quality.

## Materials and Methods

### Participants and Procedures

The online survey targeted middle-aged couples from 28 provinces in the eastern, central and western China and was conducted from January 24 to February 7, 2021. A total of 132 college student investigators from different provinces were recruited through posters in three universities in China. Prior to the baseline survey, we trained investigators on how to carry out investigation, investigation procedures and precautions. The investigators conducted a questionnaire survey on eligible couples among their friends, relatives, and neighbors. Before the investigation, the purpose of the survey, the way of answering the questionnaire and confidentiality were introduced to the surveyed couples in detail, and the existing questions were answered and informed consent was obtained. Couples who agreed to participate in the survey were provided with a number and they just need to fill in the number when answering for data matching. The research completed a survey of 1,227 middle-aged people in total. After the completion of the survey, the researchers checked all the survey data one by one and deleted the invalid data. For example, the data were not matched, the answer time was outside the extreme value of 2 standard deviations, all the questions chose the same option in all the questions, and the answers were random and regular. In the end, 1,100 valid questionnaires were retained, constituting 550 couples.

A total of 550 couples participated in the study, including 302 families living in cities and 248 in rural areas, with husbands ranging from 39 to 64 years old with an average age of 46.45 years (SD = 4.34) and wives ranging from 32 to 62 years old with an average age of 45.08 years (SD = 4.33). This study was approved by the Ethics Committee of Changchun University of Chinese Medicine (Approval No.: 2019YFC1709901).

### Measures

#### Emotion Reactivity Scale

We used the Chinese version of the Emotion Reactivity Scale to measure emotion reactivity (Nock et al., 2008). The questionnaire consists of three subscales: emotional sensitivity (10 items), emotional intensity (seven items) and emotional persistence (four items), with a total of 21 items rated on a 5-point Likert scale from 0 (not at all like me) to 4 (completely like me). The following sentence is an example of emotional sensitivity dimension items: item (5) “I tend to get very emotional very easily.” The following sentence is an example of emotional intensity dimension items: item (4) “When I’m emotionally upset, my whole body gets physically upset as well.” The following sentence is an example of emotional persistence dimension items: item (11) “When I am angry/upset, it takes me much longer than most people to calm down.” Higher scores mean the higher emotional reactivity of the individual. In the present research, the questionnaire demonstrated a good internal consistency in the data reported by husbands and wives. In this study, the husbands’ Cronbach’s alpha coefficients on the total questionnaire and each dimension questionnaire were 0.97, 0.95, 0.92, and 0.88, respectively; the wives’ Cronbach’s alpha coefficients on the total questionnaire and each dimension questionnaire were 0.97, 0.95, 0.91, and 0.87, respectively.

#### Perceived Partner Responsiveness Scale

We used the Chinese version of the Perceived Partner Responsiveness Scale (PPRS) (Reis et al., 2011). The questionnaire is a single-dimensional questionnaire with a total of 12 items. It is a 7-point Likert scale from 0 (not at all like me) to 7 (completely like me). Some instances of PPRS’s items are as follows: item (4) “My partner knows me well,” item (7) “My partner really listens to me,” and item (12) “is responsive to my needs.” Mean scores of the 12 items are calculated. The higher the score, the more response the individual perceives from their partner. In this study, the internal consistency reliability of the data reported by husbands and wives was 0.97 and 0.98, respectively.

#### Quality of Marriage Index

We used the Chinese version of the Quality of Marriage Index (QMI) (Norton, 1983). This is a single-dimensional questionnaire consisting of six items. The last item ranging from 1 (very unhappy) to 10 (very happy), and on 7-point Likert scale, ranging from 1 (not agree at all) to 7 (completely agree) for the other five items. Some instances of PPRS’s items are as follows: item (1) “We have a good marriage,” item (4) “Our marriage is strong,” and item (6) “I really feel like part of a team with my partner.” Sum scores of the six items are calculated, with higher scores reflecting better marital quality. In the data reported by husbands and wives of our study, the Cronbach’s alpha coefficient of the QMI was 0.96 and 0.97.

### Data Analysis

All data analyses were carried out using IBM SPSS Statistics (Version 21.0). Descriptive statistics were computed for all sociodemographic and study variables. Descriptive statistical analysis of husband’s and wife’s scores in Emotion Reactivity Scale (ERS), PPRS, and QMI. A paired-sample *t*-test was performed to investigate the difference between husband and wife in the scores of each variable. Pearson correlation analysis was used to investigate the correlation between ERS, PPRS and QMI scores of both spouses. In order to test the research hypothesis, AMOS (Version 24.0) was used to analyze the Actor-Partner Interdependence Model (APIM). Among them, the actor effect was the impact of the emotional reactivity of both spouses on their own marital quality, and the partner effect was the impact of the emotional reactivity of both spouses on the marital quality of the spouse. If χ^2^/df < 3, CFI > 0.9, TLI > 0.9, NFI > 0.9, AGFI > 0.9, SRMR < 0.05, RMSEA < 0.08, it meant that the model fits well. Based on the direct predictive effect of emotional reactivity on marital quality, Amos (Version 24.0) was used to test the model containing mediating variables (perceived partner responsiveness). The fitting index χ^2^/df, CFI, TLI, NFI, AGFI, SRMR, and RMSEA were also used to analyze the model and the non-significant paths were deleted. The Bootstrap method of bias correction was performed to test the mediation model.

## Results

### Descriptive Statistics

The mean and standard deviation of the research variables of middle-aged couples is shown in Table 1 separately for husbands and wives. The results of paired *t*-tests (see Table 1) showed that husbands were higher than wives in perceived partner responsiveness and marital quality, and there was no gender difference in other variables.

**TABLE 1:** Descriptive statistics of study variables for husbands (*n* = 550) and wives (*n* = 550).

	Husbands		Wives		*t*	*P*-value
	*M*	*SD*	*M*	*SD*		
ERS	16.23	15.73	16.61	15.67	–0.49	0.624
ES	7.16	7.51	7.41	7.56	–0.65	0.516
EI	5.63	5.38	5.69	5.31	–0.21	0.831
EP	3.44	3.31	3.52	3.31	–0.48	0.633
PPRS	67.07	15.02	65.54	16.19	2.50*	0.013
QMI	38.23	7.59	37.55	8.14	2.26*	0.024

M, mean; SD, standard deviation; t, paired t-test; ERS, emotion reactivity scale; ES, emotion sensitivity; EI, emotion intensity; EP, emotion persistence; PPRS, perceived partner responsiveness scale; QMI, quality of marriage index. *Significant correlations 0.05 two-tailed.

### Correlations

Intercorrelations among the study variables are ported in Table 2 separately for husbands and wives, together with correlations between couples. The results showed that husbands’ emotional reactivity was significantly negatively related with the marital quality and perceived partner responsiveness of both spouses, and the correlation coefficient was −0.25 to −0.29. Wives’ emotional reactivity was significantly negatively related with the marital quality and perceived partner responsiveness of both spouses, and the correlation coefficient was −0.25 to −0.35.

**TABLE 2:** Correlations among study variables.

	1	2	3	4	5	6	7	8	9	10	11	12
1 H ERS	–											
2 W ERS	0.32**	–										
3 H ES	0.98**	0.32**	–									
4 W ES	0.31**	0.98**	0.31**	–								
5 H EI	0.97**	0.30**	0.93**	0.29**	–							
6 W EI	0.30**	0.97**	0.30**	0.93**	0.28**	–						
7 H EP	0.94**	0.29**	0.90**	0.28**	0.88**	0.28**	–					
8 W EP	0.32**	0.93**	0.32**	0.88**	0.31**	0.86**	0.30**	–				
9 H PPRS	−0.27**	−0.25**	−0.28**	−0.24**	−0.26**	−0.25**	−0.23**	−0.22**	–			
10 W PPRS	−0.25**	−0.35**	−0.25**	−0.34**	−0.25**	−0.34**	−0.22**	−0.34**	0.58**	–		
11 H QMI	−0.29**	−0.24**	−0.30**	−0.23**	−0.27**	−0.26**	−0.25**	−0.21**	0.81**	0.53**	–	
12 W QMI	−0.25**	−0.32**	−0.25**	−0.32**	−0.24**	−0.32**	−0.20**	−0.30**	0.53**	0.88**	0.60**	–

*H, husbands; W, wives; ERS, emotion reactivity scale; ES, emotion sensitivity; EI, emotion intensity; EP, emotion persistence; PPRS, perceived partner responsiveness scale; QMI, quality of marriage index.*

**Significant correlations 0.01 two-tailed.

### Actor-Partner Interdependence Model

The APIM would be conducted to investigate the actor effect and partner effect of emotional reactivity, using emotional reactivity as a predictor variable and marital quality as an outcome variable. The model fitted well: χ^2^ = 20.478, df = 16, CFI = 0.999, TLI = 0.998, NFI = 0.995, AGFI = 0.979, SRMR = 0.012, and RMSEA = 0.015.

**FIGURE 2:**
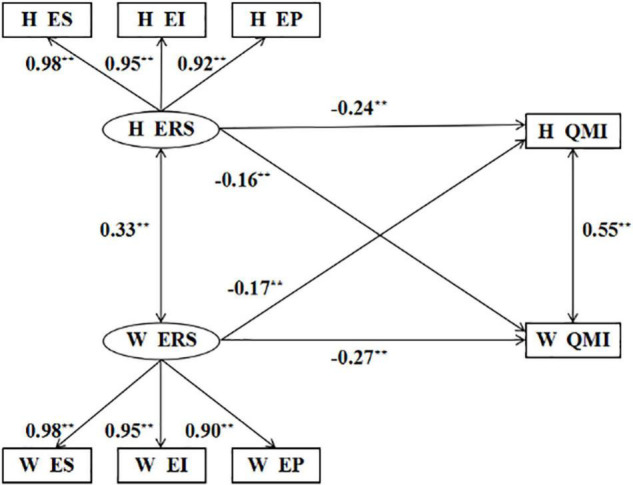
Actor-partner interdependence model. H, husbands; W, wives; ERS, emotion reactivity scale; ES, emotion sensitivity; EI, emotion intensity; EP, emotion persistence; PPRS, perceived partner responsiveness scale; QMI, quality of marriage index.**p < 0.01.

The results indicated that (see Figure 2) there was a significant negative association between the emotional reactivity and their own marital quality of husbands and wives (actor effect; husbands: β = −0.24, *p* < 0.001; wives: β = −0.16, *p* < 0.001). Additionally, we found the partner effect of emotional reactivity on marital quality was also valid. The emotional reactivity of husbands and wives was significantly negatively correlated with the quality of their partners’ marital quality (partner effect; husbands: β = −0.16, *p* < 0.001; wives: β = −0.27, *p* < 0.001).

### Mediating Effect of Perceived Partner Responsiveness

**FIGURE 3:**
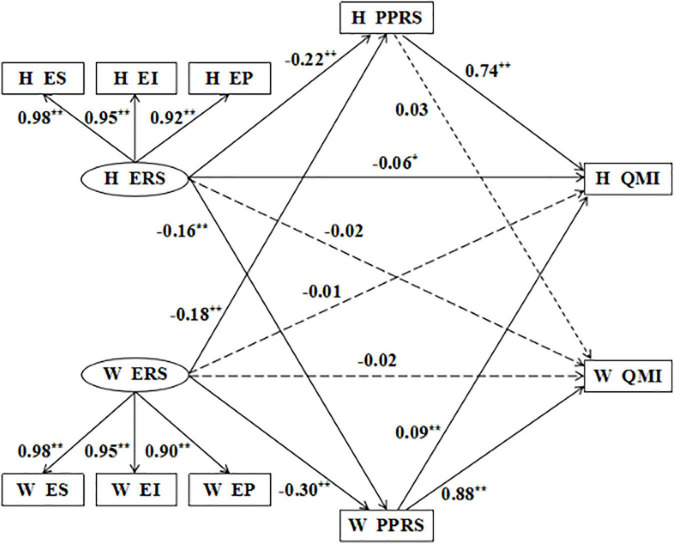
Mediating effect of perceived partner responsiveness. H, husbands; W, wives; ERS, emotion reactivity scale; ES, emotion sensitivity, EI, emotion intensity; EP, emotion persistence; PPRS, perceived partner responsiveness scale; QMI, quality of marriage index.**p < 0.01.

On the basis of the APIM, we further tested the mediating role of perceived partner responsiveness in the actor effect and partner effect of emotional reactivity on marital quality. After including perceived partner responsiveness in the model, the partner effect of husbands’ emotional reactivity on marital quality became non-significant (β = −0.02, *p* = 0.378), and the actor effect of wives’ emotional reactivity on marital quality (β = −0.02, *p* = 0.378) and partner effects (β = −0.01, *p* = 0.744) became non-significant. In addition, the path that husbands’ perceived partner responsiveness – wives’ marital quality was removed from the final model (β = 0.03, *p* = 0.298). The model fitted well after removing the non-significant path coefficient: χ^2^ = 34.274, df = 28, CFI = 0.999, TLI = 0.998, NFI = 0.994, AGFI = 0.975, SRMR = 0.013, and RMSEA = 0.020. Each path coefficient was shown in Figure 3.

As the good fitting of the model, we used the deviation correction percentile Bootstrap method to test the significance of the intermediary effect. The sample size was 5,000 and the confidence interval of 95% was calculated. As was shown in Table 3, the 95% confidence interval of each mediation path did not contain 0, showing that the three mediation paths were all significant.

**TABLE 3:** Mediating effect analysis results.

Indirect effect	β	Standard error	*p*	95% CI standardized indirect effect	
H ERS → H PPRS → H QMI	–0.17	0.040	<0.001	–0.251	–0.094
H ERS → W PPRS → H QMI	–0.015	0.009	0.024	–0.038	–0.002
H ERS → W PPRS → W QMI	–0.156	0.046	0.001	–0.254	–0.072
W ERS → H PPRS → H QMI	–0.135	0.039	<0.001	–0.214	–0.063
W ERS → W PPRS → H QMI	–0.028	0.015	0.026	–0.060	–0.003
W ERS → W PPRS → W QMI	–0.290	0.046	<0.001	–0.385	–0.200

*H, husbands; W, wives; ERS, emotion reactivity scale; ES, emotion sensitivity; EI, emotion intensity; EP, emotion persistence; PPRS, perceived partner responsiveness scale; QMI, quality of marriage index.*

## Discussion

A person’s emotional response, especially to negative emotions, can affect the individual’s mental health and social adaptation. Currently, only a few studies have focused on the impact of emotional reactivity on marital quality. In recent years, the divorce rate in China has continued to rise (Mo, 2020). How to improve the quality and stability of marriage has become a hot issue for scholars. To the best of our knowledge, the association between the emotional reactivity and marital quality among Chinese couples has never been examined. The same can also be said for the mediating role of perceived partner responsiveness. The data in this study covers most provinces in China, and can better explain the relationship between emotional reactivity, marital quality, and perceived partner responsiveness in the context of Chinese society. This study takes into account the duality of intimate relationships and takes both spouses as the research objects. It not only expounds the actor effect and partner effect of emotional reactivity on marital quality, but also analyzes the mediating mechanism of perceived partner responsiveness. This helps to better understand the interdependence of intimacy. That is, the emotional responsiveness of one party will affect the other party’s perceived partner responsiveness and marital quality. The research results have more ecological effects.

### Differences Between Husband and Wife on Marital Quality

There are differences in couples’ perceptions of marital quality. Women tend to be less satisfied with their marriage relationship, and the number of consultations on marriage and family is significantly more than that of men. In a traditional marriage relationship, the wife spends most of her time and energy on taking care of the family and children, while the husband spends more time on work, social interactions and other things outside the family (Gu, 2019). Wives are more invested in the marriage relationship and have higher expectations, while the husbands are usually the one to be taken care of and benefit more from the marriage. This will lead to the husbands’ marital quality is often higher than that of the wives’ (Shockley and Allen, 2017). In recent years, with the transformation of gender role concepts, traditional values have been replaced by modern concepts, women’s socio-economic status has been significantly improved. Women have begun to participate in political, economic, educational and other activities on a wide and equal basis, and their education level and employment rate continue to rise (Mukherjee, 2015). This may have an impact on traditional marriage relationships.

In recent years, there have been inconsistencies in the results of research on marital quality. Some studies have shown that the husbands’ marital quality is higher than that of the wife. However, some studies have found that there is no gender difference in marital quality (Bulanda et al., 2021). The emergence of inconsistent results may be related to the age and cultural background of the research subjects. Current research on the Chinese couples’ marital quality mostly focuses on newlyweds or elderly couples. While middle-aged people have received education in traditional values and were influenced by modern multiculturalism, the research on this population is relatively lacking. This study found that the marital quality of husbands is significantly higher than that of wives among middle-aged couples in China. This may be due to the fact that although women are no longer completely dependent on their husbands and have begun to participate in workplace work, women still mainly bear the responsibility of taking care of their families. The dual pressure from family and work has a destructive effect on the quality of their marriage (Huang et al., 2019).

### Actor-Partner Interdependence Model

The present study takes a dyadic approach to examine emotional reactivity in Chinese couples and its marital quality. The results of the study showed that an individual’s emotional reactivity has a negative predictive effect on their own marital quality, indicating that as the emotional reactivity increases, the individual’s marital quality showed a downward trend. This result confirms that the action effect of the individual’s emotional reactivity on marital quality is established, which is consistent with Hypothesis 1. In addition, the individual’s emotional reactivity has a negative predictive effect on the marital quality of the spouse, indicating that the partner effect of the individual’s emotional reactivity on marital quality is established, and Hypothesis 2 is valid.

This result confirms that there is more evidence about the action effect of emotional reactivity on marital quality. Previous studies have found that emotional reactivity has a low correlation with positive emotional experience, but has a high correlation with negative emotional experience (Nock et al., 2008). Individuals with high emotional responsiveness pay more attention to negative cues and are more likely to fall into negative thoughts and feelings, which will influence their own marital quality (Ye et al., 2018). Although, the previous studies have proved that emotional responsiveness negatively predicts marital quality, however, there is no research to explore the partner effect of emotional reactivity on marital quality. Significant partner effects found in the current study suggest that husbands and wives depend on each other and influence each other, and individual’s emotional reactions can affect the spouse’s marital quality. The results are consistent with the family system theory (Erdem and Safi, 2018). It shows that any individual problems will show up in the couple’s relationship (Crittenden and Dallos, 2009). This suggests that spouses need to pay attention not only to their own emotional state, but also to the emotional state of their spouse when coping with stressful events from daily life. When the spouse is experiencing negative emotions, the one should take the initiative to regulate the spouse’s emotions by means of comfort, listening, sharing, encouragement and other methods.

### Mediating Role of Perceived Partner Responsiveness

Based on the analysis of the APIM, we added the mediating variable of perceived partner responsiveness to the model. It turns out that there are differences between husbands and wives in the impact of perceived partner responsiveness on marital quality. Only the wives’ perceived partner responsiveness can positively predict their own and spouse’s marital quality, while the husbands’ perceived partner responsiveness can only positively predict their own marital quality. In other words, the husbands’ perceived partner responsiveness don’t affect the quality of the wives’ marital quality. The result is inconsistent with the existing research in the West (Reuma et al., 2016). In the context of Chinese culture, perceived partner responsiveness has different effects on men and women. Women have a high degree of emotional demand and are more involved in the process of communication between husband and wife. The husband’s positive response can promote the wife to invest more energy in the intimate relationship and help improve marital quality of both parties. In the process of couple interaction, men play more passive roles, and the wives’ active response cannot promote the husbands to invest in the relationship (Seedall and Wampler, 2016).

The study further explored the mediating effect of perceived partner responsiveness in the effect of emotional reactivity on marital quality. It was found that after adding perceived partner responsiveness to the model, the partner effect of husbands’ emotional responsiveness on marital quality became non-significant, and the actor effect and partner effect of wives’ emotional responsiveness on marital quality became non-significant. This result can explain the inconsistency of emotional responsiveness in predicting marital quality in previous studies. The Social Regulatory Cycle Theory emphasizes more on the unilateral influence of emotional regulators on emotional experiencers, but it is insufficient in explaining the influence of the emotional experiencer on the emotional regulator (Reeck et al., 2016). The results of this study provide empirical evidence for the theory of the Social Regulatory Cycle Theory in this respect. Emotional regulators may be affected by emotional experiencers, thus feeling neglected, ignored and psychological needs not being met, and affect the marital quality. Therefore, in marriage and family therapy, on the one hand, psychotherapists can use the teaching of communication skills as an intervention method to improve the marital quality by teaching couples how to express their concern, positive emotion and love for their spouses; on the other hand, psychotherapists can teach couples how to respond positively to their spouse’s psychological needs, so as to improve the perceived partner responsiveness. It can also promote the development of intimate relationships.

## Limitations and Future Directions

Limitations of this study should be noted. First of all, limited by time and funding, we adopted a cross-sectional study design, but marital quality, as a subjective feeling, is a process of dynamic change. In addition, emotional reactivity and perceived partner responsiveness may change with a person’s development, attitudes toward marriage, and changes in the relationship between the couple. Therefore, in future studies, the relationship between the three can be further explored by tracking. Secondly, the method of this study is single and only questionnaire survey is adopted, which may be affected by recall bias or social desirability, resulting in the survey results cannot accurately reflect the real marital relationship. In the future research, experimental method, interview method and other research methods can be considered. For example, it is possible to reduce the individual’s emotional reactivity through the interventions such as mindfulness trait training or compassion training. Finally, this study only examined the impact of emotional reactivity and perceived partner responsiveness on marital quality, while the couple’s personality characteristics, family socioeconomic status, attachment style and other factors also have an important impact on marital quality. It is necessary to investigate the role of different factors in the development of marital quality in the future.

## Conclusion

This study investigated the effect of emotional reactivity on marital quality and the mediating role of perceived partner responsiveness. The study found that the husbands’ perceived partner responsiveness and marital quality scores were significantly higher than those of the wives, and there was no significant difference in the emotional reactivity of the couples. The correlation analysis showed that the emotional reactivity of couples was significantly negatively correlated with perceived partner responsiveness and marital quality, while the perceived partner responsiveness was significantly positively correlated with marital quality. Based on the APIM, it is found that the emotional reactivity of both spouses is a significant negative predictor of their marital quality (actor effect). It also significantly negatively predicted the marital quality of the spouse (partner effect). The mediating effect analysis results showed that the husband’s perceived partner responsiveness played a mediating role in the emotional reactivity of the couple on the marital quality of the husband, and the wife’s perceived partner responsiveness played a mediating role in the emotional reactivity of the couple on the marital quality of the husband, while the wife’s perceived partner responsiveness played a mediating role in the emotional reactivity of the couple on the marital quality of the husband. The wife’s perceived partner responsiveness played a mediating role in the effect of the couple’s emotional reactivity on the wife’s marital quality. The results of this study contribute to a better understanding of the mechanism of emotional reactivity of couples affecting marital quality and have a guiding significance for improving marital quality.

## Data Availability Statement

The raw data supporting the conclusions of this article will be made available by the authors, without undue reservation.

## Ethics Statement

The studies involving human participants were reviewed and approved by Ethics Committee of the Affiliated Hospital of Changchun University of Chinese Medicine (Approval No.: 2019YFC1709901), Changchun University of Chinese Medicine. The patients/participants provided their written informed consent to participate in this study.

## Author Contributions

ZF contributed to the development of all aspects of this study. QY contributed to the conception, writing and data collection of the manuscript. JL participated to write the manuscript and critically modified the full text. All authors contributed to the manuscript and approved the submitted version.

## Conflict of Interest

The authors declare that the research was conducted in the absence of any commercial or financial relationships that could be construed as a potential conflict of interest.

## Publisher’s Note

All claims expressed in this article are solely those of the authors and do not necessarily represent those of their affiliated organizations, or those of the publisher, the editors and the reviewers. Any product that may be evaluated in this article, or claim that may be made by its manufacturer, is not guaranteed or endorsed by the publisher.

## References

[B1] Ali P. A. Mcgarry J. Maqsood A. (2020). Spousal role expectations and marital conflict:perspectives of men and women. *J. Interp. Viol.* 2020:0886260520966667. 10.1177/0886260520966667 33103547 PMC9092914

[B2] Alonso-Ferres M. Imami L. Slatcher R. B. (2020). Untangling the effects of partner responsiveness on health and well-being: the role of perceived control. *J. Soc. Pers. Relat*. 37 1150–1171. 10.1177/0265407519884726

[B3] Alonso-Ferres M. Righetti F. Valor-Segura I. Expósito F. (2021). How power affects emotional communication during relationship conflicts: the role of perceived partner responsiveness. *Soc. Psycholog. Person. Sci*. 12 1203–1215. 10.1177/1948550621996496

[B4] Amber A. P. Chelom E. L. David B. A. (2020). How gender differences in emotional cutoff and reactivity influence couple’s sexual and relational outcomes. *J. Sex Marital* T*her.* 2020:1800541. 10.1080/0092623X.2020.1800541 32821026

[B5] Bulanda J. R. Yamashita T. Brown J. S. (2021). Marital quality, gender, and later-life depressive symptom trajectories. *J. Women Aging* 33 122–136. 10.1080/08952841.2020.1818538 33308042

[B6] Chapman A. L. Dixon-Gordon K. L. Butler M. S. Walters K. N. (2015). Emotional reactivity to social rejection versus a frustration induction among persons with borderline personality features. *Personal. Dis. Theory Res. Treat.* 6 88–96. 10.1037/per0000101 25580675

[B7] Crittenden P. M. Dallos R. (2009). All in the family: integrating attachment and family systems theories. *Clin. Child Psychol. Psychiatry* 14 389–409. 10.1177/1359104509104048 19515755

[B8] Cundiff J. M. Smith T. W. Butner J. Critchfield K. L. Nealey-Moore J. (2015). Affiliation and control in marital interaction: interpersonal complementarity is present but Is not associated with affect or relationship quality. *Personal. Soc. Psychol. Bull.* 41 35–51. 10.1177/0146167214557002 25367005

[B9] Davies P. T. Cicchetti D. (2004). Toward an integration of family systems and developmental psychopathology approaches. *Dev. Psychopathol*. 16 477–481. 10.1017/S0954579404004626 15605621

[B10] Dell N. A. Vidovic K. R. Huang J. Pelham M. (2020). Self-Reported emotional reactivity, depression, and anxiety: gender differences among a psychiatric outpatient sample. *Social Work Res*. 44 205–214. 10.1093/swr/svaa007

[B11] Ding W. Lin X. Su J. Jiang Y. He T. (2019). The mutual influence between marital quality and child oppositional defiant disorder symptoms in Chinese families:do child and parent’s gender matter? *J. Child Family Stud.* 28 2086–2097. 10.1007/s10826-019-01408-6

[B12] Eman S. Khalid A. Nicolson R. (2019). A review of heterogeneous interpretations of emotional reactivity. *Internat. J. Emot. Educ*. 11 71–90.

[B13] Erdem G. Safi O. A. (2018). The cultural lens approach to Bowen family systems theory: contributions of family change theory. *J. Family Theory Rev*. 10 469–483. 10.1111/jftr.12258

[B14] Fowers B. J. Owenz M. B. (2010). A eudaimonic theory of marital quality. *J. Family Theory Rev*. 2 334–352. 10.1111/j.1756-2589.2010.00065.x

[B15] Frye N. Ganong L. Jensen T. Coleman M. (2020). A dyadic analysis of emotion regulation as a moderator of associations between marital conflict and marital satisfaction among first-married and remarried couples. *J. Family Issues*. 41 2328–2355. 10.1177/0192513X20935504

[B16] Gamliel K. H. Dollberg D. G. Sigal L. (2018). Relations between parents’ anxiety symptoms, marital quality, and preschoolers’ externalizing and internalizing behaviors. *J. Child Family Stud.* 27 3952–3963. 10.1007/s10826-018-1212-3

[B17] Gardner B. C. Busby D. M. Brimhall A. S. (2007). Putting emotional reactivity in its place? exploring family-of-origin influences on emotional reactivity, conflict, and satisfaction in premarital couples. *Contemp. Family Therapy* 29 113–127. 10.1007/s10591-007-9039-x

[B18] Gu C. J. (2019). Bargaining with Confucian Patriarchy: Money, culture, and gender division of labor in Taiwanese Immigrant families. *Qual. Soc*. 42 687–709. 10.1007/s11133-019-09427-x

[B19] Henry N. J. Berg C. A. Smith T. W. Florsheim P. (2007). Positive and negative characteristics of marital interaction and their association with marital satisfaction in middle-aged and older couples. *Psychol. Aging* 22 428–441. 10.1037/0882-7974.22.3.428 17874945

[B20] Huang S. L. Li R. H. Fang S. Y. Tang F. C. (2019). Well-being: its relationship with work-to-family conflict and burnout among males and females. *Internat. J. Env. Res. Public* 16:16132291. 10.3390/ijerph16132291 31261635 PMC6651233

[B21] Isanejad O. Bagheri A. (2018). Marital quality, loneliness, and internet infidelity. *Cyberpsychol. Behav. Soc. Netw*. 21 542–548. 10.1089/cyber.2017.0602 30212248

[B22] James S. L. (2015). Variation in marital quality in a national sample of divorced women. *J. Family Psychol*. 29 479–489. 10.1037/fam0000082 25915090

[B23] Lemay E. P. Clark M. S. Feeney B. C. (2007). Projection of responsiveness to needs and the construction of satisfying communal relationships. *J. Person. Soc. Psychol*. 92 834–853. 10.1037/0022-3514.92.5.834 17484608

[B24] Liu H. Cheung F. M. (2015). Testing crossover effects in an actor-partner interdependence model among Chinese dual-earner couples. *Internat. J. Psychol*. 50 106–114. 10.1002/ijop.12070 25721880

[B25] Merwin K. E. Rosen N. O. (2020). Perceived partner responsiveness moderates the associations between sexual talk and sexual and relationship well-being in individuals in long-term relationships. *J. Sex Res.* 57 351–364. 10.1080/00224499.2019.1610151 31090449

[B26] Mo L. (2020). A longitudinal study of the long-term predictors of China’s divorce rate. *Marr. Family Rev*. 56 217–240. 10.1080/01494929.2020.1712299

[B27] Mukherjee S. S. (2015). More educated and more equal? A comparative analysis of female education and employment in Japan, China and India. *Gender Educ*. 27 846–870. 10.1080/09540253.2015.1103367

[B28] Nock M. K. Wedig M. M. Holmberg E. B. Hooley J. M. (2008). The emotion reactivity scale:development, evaluation, and relation to self-injurious thoughts and behaviors. *Behav. Therapy* 39 107–116. 10.1016/j.beth.2007.05.005 18502244

[B29] Norton R. (1983). Measuring marital quality: a critical look at the dependent variable. *J. Marr. Family* 45 141–151. 10.2307/351302

[B30] O’Connor P. Izadikhah Z. Abedini S. Jackson C. J. (2018). Can deficits in emotional intelligence explain the negative relationship between abandonment schema and marital quality? *Family Relat*. 67 510–522. 10.1111/fare.12320

[B31] Olson D. H. Waldvogel L. Schlieff M. (2019). Circumplex model of marital and family systems: an update. *J. Family Theory Rev*. 11 199–211. 10.1111/jftr.12331

[B32] Overall N. C. Clark M. S. Fletcher G. J. O. Peters B. J. Chang V. T. (2020). Does expressing emotions enhance perceptual accuracy of negative emotions during relationship interactions? *Emotion* 20 353–367. 10.1037/emo0000653 31368745

[B33] Polanco-Roman L. Moore A. Tsypes A. Jacobson C. Miranda R. (2018). Emotion reactivity, comfort expressing emotions, and future suicidal ideation in emerging adults. *J. Clin. Psychol*. 74 123–135. 10.1002/jclp.22486 28493550 PMC5681888

[B34] Reeck C. Ames D. R. Ochsner K. N. (2016). The social regulation of emotion: an integrative, cross-disciplinary model. *Trends Cog. Sci.* 20 47–63. 10.1016/j.tics.2015.09.003 26564248 PMC5937233

[B35] Reis H. T. Maniaci M. R. Caprariello P. A. Eastwick P. W. Finkel E. J. (2011). Familiarity does indeed promote attraction in live interaction. *J. Person. Soc. Psychol*. 101 557–570. 10.1037/a0022885 21381850

[B36] Reuma G. Lior B. Sarah N. Ragnar A. Julia H. Eshkol R. (2016). Perceived partner responsiveness mediates the association between sexual and marital satisfaction: a daily diary study in newlywed couples. *Arch. Sex. Behav*. 45 109–120. 10.1007/s10508-014-0448-2 25680818

[B37] Rhoades K. A. Leve L. D. Harold G. T. Neiderhiser J. M. Shaw D. S. Reiss D. (2011). Longitudinal pathways from marital hostility to child anger during toddlerhood:genetic susceptibility and indirect effects via harsh parenting. *J. Family Psychol.* 25 282–291. 10.1037/a0022886 21480707 PMC3084154

[B38] Salafia E. H. B. Schaefer M. K. Haugen E. C. (2016). Connections between marital conflict and adolescent girls’ disordered eating:parent-adolescent relationship quality as a mediator. *J. Family Therapy* 38 340–368. 10.1007/s10826-013-9771-9

[B39] Seedall R. B. Wampler K. S. (2016). Couple emotional experience: effects of attachment anxiety in low and high structure couple interactions. *J. Family Therapy* 38 340–363. 10.1111/1467-6427.12113

[B40] Shapero B. G. Abramson L. Y. Alloy L. B. (2016). Emotional reactivity and internalizing symptoms:moderating role of emotion regulation. *Cogn. Therapy Res.* 40 328–340. 10.1007/s10608-015-9722-4 27231404 PMC4876867

[B41] Shockley K. M. Allen T. D. (2017). It’s not what I expected: the association between dual-earner couples’ met expectations for the division of paid and family labor and well-being. *J. Vocat. Behav*. 104 240–260. 10.1016/j.jvb.2017.11.009

[B42] Su L. Liang C. Yang X. Liu Y. (2018). Influence factors analysis of provincial divorce rate spatial distribution in China. *Disc. Dyn. Nat. Soc.* 2018:6903845. 10.1155/2018/6903845

[B43] Tasfiliz D. Selcuk E. Gunaydin G. Slatcher R. B. Corriero E. F. Ong A. D. (2018). Patterns of perceived partner responsiveness and well-being in Japan and the United States. *J. Family Psychol*. 32 355–365. 10.1037/fam0000378 29698009 PMC5922804

[B44] Ye W. Li X. Wang D. (2018). The relationship between cognitive emotion regulation of negative marital events and marital satisfaction among older people: a cross-lagged analysis. *Acta Psycholog. Sinica*. 50 426–435. 10.3724/SP.J.1041.2018.00426

[B45] Ying L. Fang L. Kwong H. (2020). Cross-lagged models of marital relationships and intergenerational conflicts during transition to parenthood:effect of patrilineal coresidence. *Family Process* 2020:12524. 10.1111/famp.12524 32023353

[B46] Yoo J. (2020). Relationships between Korean parents’ marital satisfaction, parental satisfaction, and parent–child relationship quality. *J. Soc. Person. Relat*. 37 2270–2285. 10.1177/0265407520921462

